# Were Fertile Crescent crop progenitors higher yielding than other wild species that were never domesticated?

**DOI:** 10.1111/nph.13353

**Published:** 2015-03-11

**Authors:** Catherine Preece, Alexandra Livarda, Michael Wallace, Gemma Martin, Michael Charles, Pascal‐Antoine Christin, Glynis Jones, Mark Rees, Colin P. Osborne

**Affiliations:** ^1^ Department of Animal and Plant SciencesUniversity of SheffieldSheffieldS10 2TNUK; ^2^ Department of ArchaeologyUniversity of NottinghamNottinghamNG7 2RDUK; ^3^ Department of ArchaeologyUniversity of SheffieldSheffieldS1 4ETUK; ^4^ Institute of ArchaeologyUniversity of OxfordOxfordOX1 2PGUK; ^5^ Present address: CREAFCampus de Bellaterra (UAB)Edifici C08193Cerdanyola del VallèsSpain

**Keywords:** crop progenitors, domestication, Fertile Crescent, harvest traits, origins of agriculture, seed size, yield

## Abstract

- During the origin of agriculture in the Fertile Crescent, the broad spectrum of wild plant species exploited by hunter‐gatherers narrowed dramatically. The mechanisms responsible for this specialization and the associated domestication of plants are intensely debated. We investigated why some species were domesticated rather than others, and which traits they shared.
- We tested whether the progenitors of cereal and pulse crops, grown individually, produced a higher yield and less chaff than other wild grasses and legumes, thereby maximizing the return per seed planted and minimizing processing time. We compared harvest traits of species originating from the Fertile Crescent, including those for which there is archaeological evidence of deliberate collection.
- Unexpectedly, wild crop progenitors in both families had neither higher grain yield nor, in grasses, less chaff, although they did have larger seeds. Moreover, small‐seeded grasses actually returned a higher yield relative to the mass of seeds sown. However, cereal progenitors had threefold fewer seeds per plant, representing a major difference in how seeds are packaged on plants.
- These data suggest that there was no intrinsic yield advantage to adopting large‐seeded progenitor species as crops. Explaining why Neolithic agriculture was founded on these species, therefore, remains an important unresolved challenge.

## Introduction

The Neolithic transition from a hunter‐gatherer lifestyle to an agriculture‐based existence *c*. 10 000 yr ago was a transformative event in human history. In one of the best‐documented centres of agricultural origins, the Fertile Crescent, archaeobotanical evidence indicates that the change to agricultural subsistence was associated with a significant narrowing of the spectrum of exploited species (Weiss *et al*., 2004; Willcox *et al*., 2008). Out of the dozens of wild species that were collected, processed and stored by hunter‐gatherers (Weiss *et al*., 2004; Savard *et al*., 2006; Willcox *et al*., 2008), there are eight confirmed Neolithic founder crops, seven of which are cereals and pulses (einkorn and emmer wheat, barley, chickpea, pea, lentil and bitter vetch), plus flax (Zohary *et al*., 2012). Previous work raised the possibility that further wild species may have been domesticated in the Fertile Crescent at this time, including a two‐grained type of einkorn (thought to be derived from *Triticum boeoticum* ssp. *thaoudar* or *Triticum urartu*; van Zeist, 1999) and a separate variant of emmer (possibly derived from *Triticum araraticum*; Jones *et al*., 2000), both of which are no longer cultivated. Early domestication of rye has also been suggested (Hillman, 1978) although this claim is contentious (Colledge & Conolly, 2010). The quality of the archaeobotanical evidence relating to these suggested early domestication events has been debated by Fuller *et al*. (2012b) and Abbo *et al*. (2013). Certainly, by later prehistoric periods, both rye and oats were domesticated (Zohary *et al*., 2012). Even if these plants were domesticated early, they still remain a small proportion of the available plant species. The mechanisms responsible for why certain plant species were domesticated and others were not are currently debated.

One explanation for why certain species were adopted as crops is grounded in optimal foraging theory, where foragers rank food items according to their energetic value relative to harvesting and processing costs (Smith, 1983; Stephens & Krebs, 1986; Parker & Maynard Smith, 1990). For traits commonly measured in experimental situations, total seed mass may act as a proxy for energetic value, and the quantity of chaff may relate to processing time. In this case, we would expect crop progenitors to be high‐yielding with little chaff, as these traits are predicted to increase energetic value and decrease processing costs. Recently, proponents of optimal foraging theory have strongly encouraged researchers studying the origins of agriculture to embrace methods grounded in evolutionary theory, and to use these methods to describe regional scale patterns (Gremillion *et al*., 2014a). However, this has provoked intense debate among those who believe that optimal foraging theory has failed to prove itself a useful tool for understanding the development of agriculture (Smith, 2014; Zeder, 2014; and see also Gremillion *et al*., 2014b,c).

Alternatively, crop progenitors may have been ranked lower in terms of energetic return, and additional pressures linked to demography or climate forced people to select these species despite lower energetic returns (Winterhalder & Kennett, 2006). In debates concerning the origins of agriculture in the Fertile Crescent, it has been argued by some researchers (e.g. Abbo *et al*., 2010a) that crop species were chosen and domesticated deliberately during a rapid and localized event, while others argue for a protracted transition to agriculture occurring over a wide geographic range (e.g. Fuller *et al*., 2012a). Regardless of how crop progenitors were selected, long‐term interactions between humans and plants have led to changes in both. This process can be described in terms of niche construction theory (Smith, 2011; Zeder, 2012), whereby people engineered the environment to increase the abundance of species that were highly valued; or as a coevolutionary relationship between people and the species that responded positively to exploitation (Rindos, 1984; Fuller *et al*., 2010).

Central questions in this field, therefore, are: why were certain species domesticated while others were not; and what selective pressures drove the domestication process? These can be addressed using a comparative approach by looking for traits that differ between those species that were ultimately domesticated and other wild species. Fertile Crescent cereal crop progenitors generally have large seeds relative to most of the other wild grasses (whether annual or perennial) that grow in the region (Blumler, 1998; Kluyver, 2013). This observation has led previous authors to hypothesize that large‐seeded plants are preferred over small‐seeded ones because they give a greater harvestable yield (Ladizinsky, 1975; Evans, 1993; Bar‐Yosef, 1998); have a higher ratio of grain to chaff, leading to a greater processing efficiency (Harlan, 1967); and/or are advantageous when planted in cultivated fields, as they better survive deeper burial and soil disturbance (Harlan *et al*., 1973; Fuller *et al*., 2010).

General ecological comparisons across multiple wild species show that large‐seeded species typically have a higher biomass at maturity, and greater total reproductive output than small‐seeded species (Stougaard & Xue, 2004; Rees & Venable, 2007). However, to date, there have been very few analyses of yield comparing Fertile Crescent crop progenitors with other wild plants thought to have been collected as a food source before agriculture (Harlan, 1967; Ladizinsky, 1975; Abbo *et al*., 2011), and studies often include only one or two species. A comparison across nine grass species from the Fertile Crescent showed that cereal crop progenitors had the potential to produce a higher yield than other wild grasses, but did not measure grain yield at maturity (Cunniff *et al*., 2014). If this result proves to be general across all Fertile Crescent seed crop progenitors, it provides a mechanism for why large‐seeded species could have been considered high‐value food items.

Here, we address the key question of whether crop progenitors are higher yielding than other wild species in the Fertile Crescent that could have become crops but never did. We report a comparative screening experiment of wild plants from the Fertile Crescent, which represents a significant advance on previous work in two important respects. First, where possible, we sample wild plants for which there is archaeological evidence of purposeful gathering during the time period of earliest cultivation in this region, or plants from genera that are well represented on early archaeological sites. These include the wild progenitors of the earliest domesticates. Second, we consider both wild grasses and wild legumes and, for both families, we include species with a range of seed sizes. We hypothesize that cereal and pulse crop progenitors have: higher seed yield because they produce larger plants or have greater allocation to reproduction, and greater allocation to seeds relative to chaff, than other wild species that were available during the transition to cultivation but were never adopted as crops.

## Materials and Methods

### Plant material

Two yield experiments were established, one in summer 2011 and one in summer 2013, using 24 grass species and 19 legume species in total, and between one and five accessions per species (Supporting Information Tables S1, S2). We included the progenitors of all the cereal and pulse crops known with certainty to have been domesticated at early sites in the Fertile Crescent which comprised barley (*Hordeum vulgare* ssp. *spontaneum*), einkorn wheat (*Triticum monococcum* ssp. *aegilopoides*), emmer wheat (*Triticum turgidum* ssp. *dicoccoides*), lentil (*Lens culinaris* ssp. *orientalis*), chickpea (*Cicer reticulatum*), pea (*Pisum sativum* ssp. *elatius* var. *pumilio*) and bitter vetch (*Vicia ervilia*). These species were compared with a range of other wild grass and legume species that were never domesticated, including those for which archaeobotanical evidence suggests purposeful gathering and/or storage in Neolithic settlements. We also considered species that archaeobotanical evidence suggests may have been domesticated in the Fertile Crescent, but are no longer cultivated (*T. araraticum*, of which *Triticum timopheevi* is the domesticated form, and *T. urartu*). Finally, we considered *Secale vavilovii*, a species for which the evidence of early domestication in the Fertile Crescent is equivocal, but which was certainly domesticated later (as *S. cereale*).

The full list of wild species was selected from a database compiled at the University of Sheffield, which collates all published, and some unpublished, archaeobotanical samples from Late Epipalaeolithic and Pre‐Pottery Neolithic sites throughout western Asia, building on an earlier site‐by‐site database (Connolly & Shennan, 2007). It incorporates over 3000 discrete archaeobotanical samples from 71 archaeological sites, and allowed us to identify the taxa at these sites to the lowest taxonomic level possible (sample ubiquity shown in Tables S3, S4). The archaeological context of each sample is recorded in the database, which allows identification of *in situ* storage in some cases. When archaeobotanical identifications exist only at the level of genus, an appropriate species (based on occurrence in the study region) was selected.

Species were obtained as seed from the National Plant Germplasm System (United States Department of Agriculture, Beltsville, MD, USA), the John Innes Centre Germplasm Resources Unit (Norwich, UK), the Millennium Seed Bank (Kew Gardens, Wakehurst Place, UK) and IPK Gatersleben Genebank (Stadt Seeland, Germany) using accessions collected predominantly from western Asia, including the Fertile Crescent. For grasses, two comparisons were made: one using a conservative list of crop progenitors, with *T. araraticum*,* T. urartu* and *S. vavilovii* included in the nonprogenitor category, and a second comparison where these three species were included in the crop progenitor group (labelled on figures as putative crop progenitors). Note also that *T. araraticum* and *T. urartu* were only included in the 2013 experiment. There is also some debate over the progenitor of domesticated pea, with the literature naming both *P. sativum* ssp. *elatius* and *P. sativum* ssp. *elatius* var. *pumilio* (Smykal *et al*., 2011; Zohary *et al*., 2012). Thus, both are included as progenitors in this study.

### Growth conditions

In each of the two yield experiments, the fresh mass of individual seeds was measured before planting, after the removal of outer glumes, where necessary. Legumes were scarified with sandpaper before sowing, to break dormancy. Seeds were germinated during April 2011 and March 2013, in trays containing plastic inserts with a 1 : 1 mixture of John Innes no. 2 compost (LBS Garden Warehouse, Colne, UK) and Chelford 52 washed sand (Sibelco UK Ltd, Sandbach, UK). The growth medium was saturated with water, and seeds were placed in rows to enable individuals to be identified throughout germination.

Trays were placed in a controlled‐environment growth cabinet (Conviron BDW 40, Conviron, Winnipeg, MB, Canada) with conditions set to approximate the growing season for winter annuals in the Fertile Crescent. Temperature was set to 20 : 10°C, day : night, an 8 h photoperiod and a photosynthetic photon flux density (PPFD) of 300 μmol m^−2^ s^−1^. Following germination, when seedlings reached the two‐leaf stage, they were transferred to a second cabinet at a constant temperature of 4°C (with the same light regime) for a 6–8 wk vernalization treatment to stimulate flowering. Once vernalization was completed, in July 2011 and July 2013, plants were moved to a glasshouse (Arthur Willis Environment Centre, University of Sheffield, UK) and individuals were transferred into 11 l square pots (20 × 20 × 25 cm), again with a 1 : 1 mixture of John Innes no. 2 compost and Chelford 52 washed sand. These large pots greatly exceeded the recommended minimum soil volume (1 l for each 2 g of dry plant mass) required to avoid restriction of root growth in comparative experiments (Poorter *et al*., 2012). The temperature was maintained at 24 : 15°C, day : night, and the glasshouse was naturally sunlit during the high‐light conditions of summertime.

### Experimental design

The experiment in 2011 was split between three glasshouse rooms, with a randomized block design and 20 blocks in total (six or seven blocks per room). Each block contained one individual of each species where possible. Plants were watered three times per week and given Long Ashton nutrient solution (50% concentration) at two points during the growing period. Plant mortality and flowering were tracked during the experiment, focusing on the first flowering date. Whilst the vast majority of species can self‐pollinate, the two *Secale* species (*S. strictum* and *S. vavilovii*) are self‐incompatible, and cross‐pollination was therefore carried out manually using a paintbrush. Seeds were allowed to develop to maturity and, to prevent dispersal of wild grass seed (through natural shattering of the brittle rachis), translucent, cellophane crossing bags (Focus Packaging and Design Ltd, Worlaby, UK) were used on a subset of spikes (> five per plant) for each plant. For wild legume species, seeds were harvested as soon as they were ripe (before shattering). For the experiment in 2013, two glasshouse rooms were used, again with a randomized block design and 10 blocks in total, with one individual of each species per block. All other aspects of the experimental setup were the same as in the first experiment.

### Trait measurements

In the first experiment, when plants reached maturity, after 2 months of growth in September 2011, maximum total plant height for grasses was measured, with the plant fully extended. For legumes, the longest shoot length was measured, again fully extended. Also at this time, tiller number was counted for all grass species, and then above‐ground biomass was harvested, and divided into vegetative and reproductive tissues. The harvested biomass was oven‐dried at 40°C for 3 d before weighing. The components of seed yield were assessed by measuring the number of seeds and mean seed size for a subset of the spikes/pods of each plant. The total number of spikes or pods per plant was also counted and used to calculate the total seed mass per plant (mean mass of seed per spike or pod × number of spikes or pods), referred to as the total seed yield. Also measured for grasses were the mass of seeds per spike, the seed number per spike, the number of spikes per plant, and the mass of chaff per seed (shown as a percentage of the unthreshed grain). Time to flowering is reported as the Julian date of first flower. We distinguish between individual seed mass at the time of sowing (hereafter simply called individual seed mass), and individual seed mass at harvest, which is the mean individual mass of seeds produced by each plant at the end of the experiment. In the second experiment, seed yield and total above‐ground biomass were measured using the same methods.

### Phylogeny

Comparisons between the species were performed in a phylogenetic context (see the following section on statistical analyses). This approach was taken in order to account for the nonindependence of related species without relying on taxonomic classifications of species. This is because taxonomy does not always follow phylogenetic relationships, meaning that a comparative analysis based on genus would have been inappropriate in some cases. In particular, in the grasses, phylogenetic analyses have revealed that the *Triticum* and *Aegilops* species cannot be distinguished at the genus level, with one branch of the phylogenetic tree including *Aegilops speltoides*,* T. araraticum* and *T. dicoccoides*, and another containing *Aegilops crassa*,* Aegilops tauschii*,* T. urartu* and *T. monococcum* ssp. *aegilopoides* (Petersen *et al*., 2006; Marcussen *et al*., 2014). In the legumes there is a similar situation with *V. ervilia*, which comprises a sister group to other *Vicia* species (Oskoueiyan *et al*., 2010). These relationships were confirmed by our phylogenies.

In order to apply these approaches, separate phylogenetic trees were constructed for the grasses and legumes (Figs S1, S2). For legumes, the phylogeny was based on the plastid marker *trnKmatK* and, for grasses, on the plastid markers *ndhF* and *trnKmatK*. Sequences were first retrieved from GenBank for the species of interest and a number of closely related species not used in the screening experiments. For species not available in GenBank, genomic DNA was isolated using the DNeasy Plant Mini Kit (Qiagen). The plastid markers were then PCR‐amplified and sequenced using published protocols and primers, following Christin *et al*. (2011) for legumes and the Grass Phylogeny Working Group II (2012) for grasses. The markers were then independently aligned for the grasses and legumes using MUSCLE (Edgar, 2004), and the alignments were manually refined. These sequence data have been submitted to GenBank under the accession numbers KM487280–KM487296. Ultrametric phylogenetic trees were then obtained through Bayesian inference as implemented in BEAST (Bayesian evolutionary analysis by sampling trees; Drummond & Rambaut, 2007). The substitution model was set to a GTR + G + I, without partition. A relaxed log‐normal molecular clock was used, with a Yule speciation prior. The age of the root of each tree was set to an arbitrary value of 10. The tree was rooted by forcing the monophyly of both the outgroup and ingroup. For grasses, the outgroup of the clade of interest (the Pooideae subfamily) is the Nardeae (*Lygeum* and *Nardus*; Grass Phylogeny Working Group II, 2012). For legumes, the subfamily Cesalpinioideae, represented by *Lemuropisum* and *Pterogyne*, was used as the outgroup (Wojciechowski *et al*., 2004). For each dataset, two different analyses were run, each for 10 000 000 generations, sampling a tree every 1000 generations. Convergence, effective sample size (ESS), and appropriateness of the burn‐in period were assessed with Tracer (Rambaut & Drummond, 2007). The burn‐in period was set to 1000 000 generations, and the maximum‐probability tree of all 18 000 000 sampled trees was selected. Age nodes were plotted as medians over the sampled trees.

### Statistical analyses

Data were analysed in a phylogenetic context using linear mixed‐effects models in R, using the lmekin function in the coxme package, and generalized least‐squares, using the pgls function in the caper package. Both methods were used, as each has different advantages. The lmekin analysis is preferable as it uses all the data rather than summary statistics (e.g. mean values as in the pgls analysis). However, inference for mixed models is more complicated and *P*‐values for fixed effects are often too small. We therefore also used pgls models applied to the species means, as statistical inference in this case is more straightforward and the *P*‐values are more conservative. In all cases, we quote the most conservative *P*‐values (usually from the pgls analysis), but calculate effect sizes from the mixed model. In both models, we estimated Pagel's *λ* to account for variation between species not related to phylogeny. The difference in plant traits between crop progenitors and other wild species was tested as a fixed effect in both analyses. The relationship between total yield and individual seed mass was also tested using the same statistical methods. When data were combined from the two experiments, an additional random effect for experiment was included in the mixed model analysis.

An example of the model used in the lmekin analysis is as follows: lmekin (ln.seed.mass ~ status + (1|species) + (1|experiment), data = grass, varlist = list (list (spp.var,var.cov.tree))). An example of the model used in the pgls analysis is as follows: pgls (ln.yield.g ~ status, data = dat, lambda = ‘ML’). For lmekin analyses on grasses, the number of replicates per species was 11–20 for traits measured in 2011 only, and nine to 30 for traits measured in both years. For legumes, the replication was 10–20 for the 2011 data, and nine to 29 for both years. In the pgls model, we averaged the data for each species over both experiments; therefore the number of replicates is equal to the number of species. For grasses this was 15 in the 2011 experiment and 24 for both years together, and for legumes this was nine in 2011 and 19 for both together. There was no significant effect of accession, and therefore this was not included in the models. Log_e_ transformations were applied to all variables. All comparisons were tested at the 0.05 significance level.

## Results

**Figure 1:**
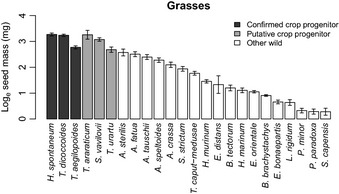
The mass of individual seeds sown for grass species, shown as the log_e_ of seed mass (mg). Data are shown in categories of domestication status (confirmed founder crop progenitors, putative crop progenitors and other wild species) in descending order of individual seed mass. Species are *Hordeum vulgare* ssp. *spontaneum*,* Triticum turgidum* ssp. *dicoccoides*,* Triticum monococcum* ssp. *aegilopoides*,* Triticum araraticum*,* Secale vavilovii*,* Triticum urartu*,* Avena sterilis*,* Avena fatua*,* Aegilops tauschii*,* Aegilops speltoides*,* Aegilops crassa*,* Secale strictum*,* Taeniatherum caput‐medusae*,* Hordeum murinum* ssp. *glaucum*,* Eremopyrum distans*,* Bromus tectorum*,* Hordeum marinum* ssp. *gussoneanum*,* Eremopyrum orientale*,* Bromus brachystachys*,* Eremopyrum bonaepartis*,* Lolium rigidum*,* Phalaris minor*,* Phalaris paradoxa* and *Stipa capensis*. Error bars show ± 1 SEM. Crop progenitors have larger seed mass both when the conservative list of confirmed progenitors is used (*F*_1,22_ = 7.0, *P* < 0.05) and when the putative progenitors are included (*F*_1,22_ = 43.8, *P* < 0.001).

**Figure 2:**
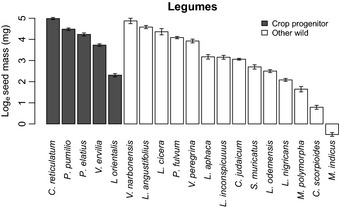
The mass of individual seeds sown for legume species, shown as the log_e_ of seed mass (mg). Data are shown in categories of domestication status (crop progenitors and other wild species) in descending order of individual seed mass. Species are *Cicer reticulatum*,* Pisum sativum* ssp. *elatius* var. *pumilio*,* P. sativum* ssp. *elatius*,* Vicia ervilia*,* Lens culinaris* ssp. *orientalis*,* Vicia narbonensis*,* Lupinus angustifolius*,* Lathyrus cicera*,* Pisum fulvum*,* Vicia peregrina*,* Lathyrus aphaca*,* Lathyrus inconspicuus*,* Cicer judaicum*,* Scorpiurus muricatus*,* Lens culinaris* ssp. *odemensis*,* Lens nigricans*,* Medicago polymorpha*,* Coronilla scorpioides* and *Melilotus indicus*. Error bars show ± 1 SEM.

Cereal crop progenitors had, on average, larger seeds (individual seed mass) than the sample of wild grass species that were never domesticated. This was true for both the conservative list of crop progenitors (2.7 times larger, 95% CI: 1.4–4.9, *P *<* *0.05) and the longer list, including possible additional early crop progenitors (3.4 times larger, 95% CI: 2.4–4.8, *P *<* *0.001) (Table 1; Fig. 1). The same pattern was observed for pulse crop progenitors and the sample of other wild legume species, although the difference was nonsignificant (1.1 times larger, ns) (Table 1; Fig. 2). There was a greater range of individual seed mass amongst the pulse crop progenitors (Fig. 2).

**Table 1:** Differences in reproductive and vegetative traits between crop progenitor and other wild species of grasses and legumes (mixed‐effects model with a genetic random effect and additional random effect of experiment when necessary)

Trait	Grasses		Legumes	
	Effect size	*F*_df_, *P*	Effect size	*F*_df_, *P*
Individual seed mass (sown)	Progenitors 2.8 times larger	7.0_1,22_, < 0.05	No effect	ns
Total seed yield	No effect	ns	No effect	ns
Individual seed mass at harvest	Progenitors 2.3 times larger	6.0_1,13_, < 0.05	No effect	ns
Seed number per spike	No effect	ns	–	–
Spike mass	No effect	ns	–	–
Spike/pod number	Wild 2.2 times greater	19.8_1,13_, < 0.001	No effect	ns
Seed number per plant	Wild 4.6 times greater	12.5_1,13_, < 0.01	–	–
Biomass	No effect	ns	No effect	ns
Height	No effect	ns	No effect	ns
Reproductive/vegetative biomass ratio	No effect	ns	No effect	ns
Flowering date	No effect	ns	No effect	ns
% Chaff	No effect	ns	No effect	ns

The results for the grasses are those using the list of three confirmed cereal progenitors. *P* < 0.05 indicates a significant difference between crop progenitors and other wild species. ns, no significant difference; –, trait that was not measured for legumes.

**Figure 3:**
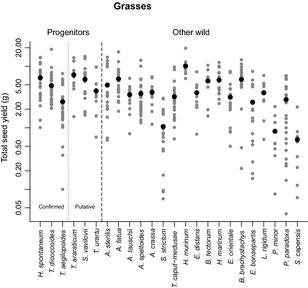
Total seed yield (g) of cereal crop progenitors and other wild grasses that were potentially gathered before the transition to agriculture. Crop progenitors are separated into confirmed and putative progenitors. Data shown are for all individuals (grey circles) and the species mean (black circles), and species are in descending order of individual seed mass at sowing within each group. Species are *Hordeum vulgare* ssp. *spontaneum*,* Triticum turgidum* ssp. *dicoccoides*,* Triticum monococcum* ssp. *aegilopoides*,* Triticum araraticum*,* Secale vavilovii*,* Triticum urartu*,* Avena sterilis*,* Avena fatua*,* Aegilops tauschii*,* Aegilops speltoides*,* Aegilops crassa*,* Secale strictum*,* Taeniatherum caput‐medusae*,* Hordeum murinum* ssp. *glaucum*,* Eremopyrum distans*,* Bromus tectorum*,* Hordeum marinum* ssp. *gussoneanum*,* Eremopyrum orientale*,* Bromus brachystachys*,* Eremopyrum bonaepartis*,* Lolium rigidum*,* Phalaris minor*,* Phalaris paradoxa* and *Stipa capensis*. There is no difference in total yield between the two groups of species.

**Figure 4:**
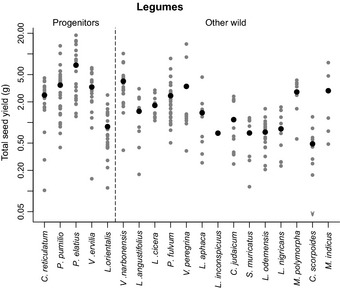
Total seed yield (g) of pulse crop progenitors and other wild legumes that were potentially gathered before the transition to agriculture. Data shown are for all individuals (grey circles) and the species mean (black circles), and species are in descending order of individual seed mass at sowing within each group. Species are *Cicer reticulatum*,* Pisum sativum* ssp. *elatius* var. *pumilio*,* P. sativum* ssp. *elatius*,* Vicia ervilia*,* Lens culinaris* ssp. *orientalis*,* Vicia narbonensis*,* Lupinus angustifolius*,* Lathyrus cicera*,* Pisum fulvum*,* Vicia peregrina*,* Lathyrus aphaca*,* Lathyrus inconspicuus*,* Cicer judaicum*,* Scorpiurus muricatus*,* Lens culinaris* ssp. *odemensis*,* Lens nigricans*,* Medicago polymorpha*,* Coronilla scorpioides*,* Melilotus indicus*. There is no difference in total yield between the two groups of species.

**Figure 5:**
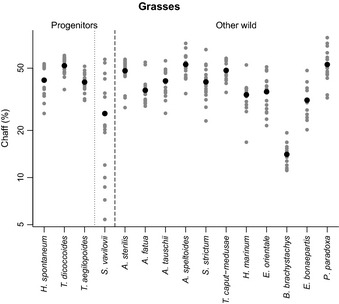
Chaff of grasses, shown as a percentage of the total grain and chaff mass. Crop progenitors are separated into confirmed and putative progenitors. Data shown are for all individuals (grey circles) and the species mean (black circles), and species are in order of descending order of individual seed mass at sowing within each group. Species are *Hordeum vulgare* ssp. *spontaneum*,* Triticum turgidum* ssp. *dicoccoides*,* Triticum monococcum* ssp. *aegilopoides*,* Secale vavilovii*,* Avena sterilis*,* Avena fatua*,* Aegilops tauschii*,* Aegilops speltoides*,* Secale strictum*,* Taeniatherum caput‐medusae*,* Hordeum marinum* ssp. *gussoneanum*,* Eremopyrum orientale*,* Bromus brachystachys*,* Eremopyrum bonaepartis*,* Phalaris paradoxa*. There is no difference in allocation to chaff between the two groups of species.

A comparison of reproductive and vegetative traits between crop progenitors and other wild plant species showed no difference in the total seed yield per plant for either grasses or legumes (Figs 3, 4). When compared across species, total seed yield was not correlated with individual seed mass for grasses, and there were two small‐seeded grasses that had high total seed yield – *Hordeum murinum* ssp. *glaucum* and *Bromus brachystachys*. However, there was a strong positive correlation between these two traits across the legume species (*P *<* *0.001) (Fig. S3). There was also no difference between crop progenitors and other wild species in allocation to chaff in grasses (Fig. 5) or allocation to reproductive biomass in either family (Table 1). However, for grasses, there were differences in the components of yield, and these varied depending on which founder crop progenitors were included. Hence, using the shorter, conservative list, crop progenitors had a greater individual seed mass at harvest (3.1 times larger, 95% CI: 1.4–7.3, *P *<* *0.05), fewer spikes per plant (other wild species have 2.8× more, 95% CI: 1.7–4.4, *P *<* *0.001) and fewer seeds per plant (other wild species have three times as many, 95% CI: 1.5–5.7, *P *<* *0.01) (Table 1). When rye was also included as a founder crop progenitor (we did not collect data for *T. araraticum* and *T. urartu* in 2011), progenitors again had a larger individual seed mass at harvest (3.5 times larger, 95% CI: 2.1–5.9, *P *<* *0.0001) and fewer seeds per plant (other wild species have 2.5 times as many, 95% CI: 1.2–5.2, *P *<* *0.05). However, there was no difference in spike number, and instead spike mass was greater in crop progenitors (3.1 times larger, 95% CI: 1.8–5.4, *P *<* *0.001). The variability of these traits is shown in Figs S4, S5.

There were no significant differences in total above‐ground biomass, height or time to flowering between crop progenitors and other wild species, in either family. For cereals, there was a weak positive correlation between the mass of individual seeds sown and above‐ground biomass (*P *<* *0.05) but not with height. For the legumes in our comparison, there were no significant differences in any traits between progenitors and other wild species (Table 1). However, there were positive correlations between the mass of individual seeds sown and above‐ground biomass (*P *<* *0.001) and height (*P *<* *0.05).

## Discussion

This study is the first to compare a comprehensive breakdown of yield components among a broad sample of grain species that were potentially gathered at pre‐agricultural sites in the Fertile Crescent. It is also the first to investigate whether crop progenitors could produce a clear yield advantage over other wild species available in the local species pool. Our experimental data do not support the often‐assumed yield advantage of crop progenitor species. For legumes, in general, we found no trait differences between crop progenitors and other wild species. However, our data highlight a number of differences in reproductive and vegetative traits between cereal crop progenitors and other wild grass species. These traits may have been important for the foraging decisions made by gatherers during the transition to agriculture, and hence in filtering which wild species were taken into cultivation and domesticated, and which were abandoned as food plants.

Previous work on grasses (Blumler, 1998) has shown that cereal crop progenitors had larger seeds than most other wild grasses. Pulse crop progenitors, however, are not exclusively the largest seeded legume species available in the Fertile Crescent. Our experiments found no difference in the total seed yield per plant between crop progenitors and other wild species in either family. Although the cereal crop progenitors were larger‐seeded than the other wild grass species, there was no relationship between yield and seed size in comparisons across species. Some of the small‐seeded wild grass species were therefore capable of producing seed yields equivalent to those of crop progenitors. The pulse crop progenitors were not significantly larger‐seeded than the other wild legumes in the experiments, but in this case there was a yield–seed size relationship. Overall, then, there was no difference in total seed yield between crop progenitors and other wild species, but this arose for different reasons in the two plant families. Additionally, there was no difference in allocation to reproductive biomass, the percentage of chaff in grasses, or the number of pods in legumes, which also confounds previous expectations about the selection of crop progenitors being related to shorter processing times.

Our results strongly challenge the view that crop progenitors have greater value to gatherers because they produce a higher yield per plant (Ladizinsky, 1975; Evans, 1993; Bar‐Yosef, 1998) or, for grasses, that they are easier to process because their ears contain a lower proportion of chaff (Harlan, 1967). Whilst there are examples of previous studies that have cast doubt on the central importance of yield maximization (Abbo *et al*., 2010b), the majority of explanations for why certain species became domesticated do assume higher yields, or at least higher calorific values. Our results provide an important, novel contribution because we quantify seed yield in an experimental setting, for a large number of species; and we specifically test the difference between crop progenitors and other wild species (rather than domesticated species), which is the critical comparison to make when trying to understand selection of crop progenitors.

To understand the implications of yield being independent of individual seed mass for the selection of crop species, we need to consider two harvesting and storage scenarios. In the first, we imagine that a constant number of seeds were stored for use the following year. In this scenario, because total seed yield is independent of individual seed mass, there is no inherent advantage to storing and using large‐seeded species. In fact, it is more efficient to use small‐seeded species, as a lower fraction of harvested seed mass needs to be stored. In the second scenario, we imagine that a constant mass, or volume, of seeds is stored for sowing in the following year. In this case, the total seed yield from all seeds sown is inversely proportional to individual seed mass. So there is an enormous advantage to using small‐seeded species (halving the mass of seeds sown doubles the seed yield).

The fact that smaller‐seeded species were overwhelmingly not domesticated in the Fertile Crescent, despite their potentially high yield per mass of seeds sown, suggests that factors other than yield were involved in the selection of large‐seeded species as crops. Crop selection may be linked to traits that affect how many plants can grow in a given area as, from the viewpoint of an early cultivator, yield may be more important at the stand level rather than for individual plants. It has frequently been shown in crops that, when the number of plants growing in a given area is increased, there is an increase in total yield (e.g. Nerson, 1980; Loss *et al*., 1998; Spink *et al*., 2000), but only up to a certain density of plants. After that density (which varies depending on the species), there are no further gains in yield, owing to the phenomenon of constant final yield (Harper, 1977; Weiner & Freckleton, 2010), whereby yield is stabilized through mechanisms such as greater tiller death (Kirby, 1967) or a reduction in the number of seeds produced by each plant (Spink *et al*., 2000; El‐Zeadani *et al*., 2014). Our data show that large‐seeded cereal crop progenitors have fewer seeds per plant and fewer spikes per plant than other wild species, whilst maintaining the same total seed yield per plant. A similar pattern emerged if a longer list of potential founder crop progenitors was considered. These traits could contribute towards greater yield per unit area, if the production of fewer flowering tillers allows individual plants to grow closer together.

In our experiment, we investigated yield at the individual level, as this enabled us to compare more species and have more replicates than if we had been trying to establish a field experiment. The density of individual plants among pots was 25 individuals m^–2^, which is far lower than modern‐day seeding rates of *c*. 300 seeds m^–2^ (Spink *et al*., 2000), and is likely to be lower than the natural densities of these species in the wild. Growth at a higher plant density would certainly change the yield, and components of yield, of individual plants. We must therefore be careful not to assume that the traits of individually grown plants correspond exactly with traits of plants grown in competition (Neytcheva & Aarssen, 2008). Indeed, there is a requirement for further research on the impacts of density on intraspecific competition within crop progenitors compared with other wild species.

Selection of crop progenitors may also be related to how easy it is to harvest or handle the seeds, as this has been shown to be true of domesticated species (Evans, 1993; Tzarfati *et al*., 2013; Abbo *et al*., 2014), which implies a deliberate human choice, or to other traits not dependent on deliberate human selection related to an unconscious process. Although we found no differences in the proportion of chaff in cereal seeds, the differences we found relating to the number and size of spikes and seeds per plant may have contributed to more efficient processing of cereal crop progenitors, because seeds are concentrated in fewer spikes.

Overall, our data do not support the view that the origins of agriculture can be explained within the optimal foraging theory framework. However, caution must be exercised in extrapolating from our results from individual plants growing in pots within a glasshouse to the situation of plant stands growing in the field. Finally, further alternative hypotheses about the selection of crop progenitors may consider yield stability (rather than yield maximization) (Abbo *et al*., 2010b) – implying intentional human decisions – as well as traits that may have allowed crop progenitors to outcompete other plant species in anthropogenic environments during the transition to agriculture (Cunniff *et al*., 2014) – a potential unintended consequence of plant collection and cultivation. Future studies should aim to test these hypotheses experimentally, ideally in field experiments in western Asian environments.

Our experimental screening of wild grass and legume species that could have been gathered in the Fertile Crescent during the transition to agriculture demonstrates that, contrary to expectations, crop progenitors do not inherently produce a greater total seed yield than other wild species. We also found no evidence that grasses produce a lower ratio of chaff to seed. Instead, for grasses but not legumes, crop progenitors were distinguished from other wild species by a suite of other harvest traits related to the packaging of seeds in spikes, and these coincided with a large difference in individual seed mass. Assumptions about the value of deliberately selecting larger‐seeded species, in order to achieve higher yields, may need to be reconsidered if we are to fully understand the origins of agriculture.

## Supporting information

Please note: Wiley Blackwell are not responsible for the content or functionality of any supporting information supplied by the authors. Any queries (other than missing material) should be directed to the *New Phytologist* Central Office.

**Fig. S1** Grass phylogeny for the 24 species used in our experiments.
**Fig. S2** Legume phylogeny for the 19 species used in our experiments.
**Fig. S3** Relationship between total seed yield and individual seed mass.
**Fig. S4** Variability of traits relating to total seed yield in grasses.
**Fig. S5** Variability of traits relating to total seed yield in legumes.
**Table S1** List of grass accessions used, including accession number on the Germplasm Resources Information System (GRIN) database, country of origin and mean individual seed mass in mg (± 1SE)
**Table S2** List of legume accessions used, including accession number on the Germplasm Resources Information System (GRIN) database, country of origin and mean individual seed mass in mg (± 1SE)
**Table S3** Sample ubiquity (number of samples in which taxon occurs) list for the grass taxa used in this study, acquired from the archaeobotanical database
**Table S4** Sample ubiquity (number of samples in which taxon occurs) list for the legume taxa used in this study, acquired from the archaeobotanical database
