# Supplementary material for: Were Fertile Crescent crop progenitors higher yielding than other wild species that were never domesticated?
Source: New Phytol. 2015 Mar 11;207(3):905–13. doi: 10.1111/nph.13353 (PMC4949991; doi:10.1111/nph.13353)
Supplement: Supplementary file 1 — Fig. S1 Grass phylogeny for the 24 species used in our experiments. Fig. S2 Legume phylogeny for the 19 species used in our experiments. Fig. S3 Relationship between total seed yield and individual seed mass. Fig. S4 Variability of traits relating to total seed yield in grasses. Fig. S5 Variability of traits relating to total seed yield in legumes. Table S1 List of grass accessions used, including accession number on the Germplasm Resources Information System (GRIN) database, country of origin and mean individual seed mass in mg (± 1SE) Table S2 List of legume accessions used, including accession number on the Germplasm Resources Information System (GRIN) database, country of origin and mean individual seed mass in mg (± 1SE) Table S3 Sample ubiquity (number of samples in which taxon occurs) list for the grass taxa used in this study, acquired from the archaeobotanical database Table S4 Sample ubiquity (number of samples in which taxon occurs) list for the legume taxa used in this study, acquired from the archaeobotanical database [file NPH-207-905-s001.pdf]

***New Phytologist* Supporting Information Figs S1–S5 and Tables S1–S4**

Article title: **Were Fertile Crescent crop progenitors higher yielding than other wild species that were never domesticated?**

Authors: Catherine Preece, Alexandra Livarda, Michael Wallace, Michael Charles, Gemma Martin, Pascal-Antoine Christin, Glynis Jones, Mark Rees and Colin P. Osborne

Article acceptance date: 31 January 2015

The following Supporting Information is available for this article:

**Fig. S1** Grass phylogeny for the 24 species used in our experiments.

**Fig. S2** Legume phylogeny for the 19 species used in our experiments.

**Fig. S3** Relationship between total seed yield and individual seed mass.

**Fig. S4** Variability of traits relating to total seed yield in grasses.

**Fig. S5** Variability of traits relating to total seed yield in legumes.

**Table S1** List of grass accessions used, including accession number on the Germplasm Resources Information System (GRIN) database, country of origin and mean individual seed mass in mg (± 1SE)

**Table S2** List of legume accessions used, including accession number on the Germplasm Resources Information System (GRIN) database, country of origin and mean individual seed mass in mg (± 1SE)

**Table S3** Sample ubiquity (number of samples in which taxon occurs) list for the grass taxa used in this study, acquired from the archaeobotanical database

**Table S4** Sample ubiquity (number of samples in which taxon occurs) list for the legume taxa used in this study, acquired from the archaeobotanical database

**Table S1** List of grass accessions used, including the seed bank it was obtained from, country of origin and mean individual seed mass in mg (± 1SE)

| Species | Group | Seed bank | Accession numbers | Country of origin | Mean seed mass (mg) (± 1SE) | Experiment |
|---|---|---|---|---|---|---|
| *Aegilops crassa* Boiss. | Other wild | GRIN | PI 487286 | Jordan | 13.77 ± 1.28 | 2013 |
| | | GRIN | PI 219863 | Iraq | 15.36 ± 1.37 | 2013 |
| | | GRIN | PI 245725 | Turkey | 19.50 ± 1.00 | 2013 |
| *Aegilops speltoides* Tausch | Other wild | GRIN | PI 170204 | Turkey | 6.81 ± 0.27 | 2011 |
| | | GRIN | PI 487231 | Syria | 6.26 ± 0.57 | 2011 |
| | | GRIN | PI 487234 | Syria | 6.58 ± 1.19 | 2013 |
| | | JIC | 2140006 | Israel | 8.56 ± 0.64 | 2013 |
| | | JIC | 2140015 | Turkey | 9.77 ± 1.74 | 2013 |
| *Aegilops tauschii* Coss. | Other wild | GRIN | PI 486275 | Turkey | 8.68 ± 0.37 | 2011 |
| | | GRIN | PI 511370 | Iran | 16.11 ± 0.90 | 2011 |
| *Avena fatua* L. | Other wild | GRIN | PI 126259 | Afghanistan | 16.06 ± 0.68 | 2011 |
| | | GRIN | PI 173584 | Turkey | 10.85 ± 0.60 | 2011 |
| *Avena sterilis* L. | Other wild | GRIN | PI 326955 | Israel | 11.18 ± 0.59 | 2011 |
| | | GRIN | PI 134251 | Afghanistan | 22.32 ± 0.19 | 2011 |
| | | GRIN | PI 309424 | Israel | 13.64 ± 1.31 | 2011 |
| *Bromus brachystachys* Hornung | Other wild | GRIN | PI 220582 | Afghanistan | 2.49 ± 0.13 | 2011 & 2013 |
| | | GRIN | PI 229598 | Iran | 2.47 ± 0.15 | 2013 |
| *Bromus tectorum* L. subsp. *lucidus* Sales | Other wild | GRIN | PI 317422 | Afghanistan | 3.39 ± 0.20 | 2013 |
| *Eremopyrum bonaepartis* (Spreng.) Nevski | Other wild | GRIN | PI 227343 | Iran | 1.78 ± 0.07 | 2011 & 2013 |
| | | GRIN | PI 227345 | Iran | 3.08 ± 0.10 | 2011 |
| | | IPK | GRA 791 | Iran | 2.53 ± 0.14 | 2013 |
| | | GRIN | PI 203442 | Turkey | 2.29 ± 0.27 | 2013 |
| *Eremopyrum distans* (K. Koch) Nevski | Other wild | JIC | 6060001 | Afghanistan | 2.91 ± 0.09 | 2013 |
| | | JIC | 6060002 | Afghanistan | 3.43 ± 0.52 | 2013 |
| *Eremopyrum orientale* (L.) | Other wild | GRIN | PI 219963 | Afghanistan | 2.79 ± 0.06 | 2011 |

| | | | | | | |
|---|---|---|---|---|---|---|
| Jaub. & Spach | | GRIN | PI 229425 | Iran | 3.05 ± 0.08 | 2011 |
| *Hordeum marinum* subsp. | Other wild | GRIN | PI 204582 | Turkey | 3.67 ± 0.25 | 2011 |
| *gussoneanum* (Parl.) Thell. | | GRIN | PI 220522 | Afghanistan | 2.09 ± 0.18 | 2011 |
| *Hordeum murinum* subsp. | Other wild | GRIN | PI 401361 | Iran | 3.69 ± 0.54 | 2013 |
| *glaucum* (Steud.) Tzvelev | | GRIN | PI 204874 | Turkey | 4.69 ± 0.38 | 2013 |
| | | GRIN | PI 223371 | Israel | 3.48 ± 0.36 | 2013 |
| *Hordeum vulgare* L. subsp. | Confirmed | GRIN | PI 282628 | Israel | 19.37 ± 1.34 | 2011 |
| *spontaneum* (K. Koch) Thell. | Progenitor | GRIN | PI 466114 | Syria | 26.76 ± 1.08 | 2011 |
| | | GRIN | PI 236387 | Syria | 28.67 ± 0.74 | 2013 |
| | | GRIN | PI 354944 | Israel | 33.27 ± 2.30 | 2013 |
| | | GRIN | HOR2688 | Iran | 25.45 ± 2.18 | 2013 |
| *Lolium rigidum* Gaudin | Other wild | GRIN | PI 254899 | Iraq | 2.20 ± 0.19 | 2013 |
| | | GRIN | PI 545595 | Turkey | 1.29 ± 0.09 | 2013 |
| *Phalaris minor* Retz. | Other wild | Kew | 113968 | Jordan | 1.43 ± 0.09 | 2013 |
| | | GRIN | PI 170628 | Turkey | 1.24 ± 0.08 | 2013 |
| | | GRIN | PI 226519 | Iran | 2.05 ± 0.12 | 2013 |
| *Phalaris paradoxa* L. | Other wild | GRIN | PI 202684 | Turkey | 2.24 ± 0.29 | 2011 |
| | | GRIN | PI 380964 | Iran | 1.76 ± 0.04 | 2011 & 2013 |
| | | GRIN | PI 233268 | Israel | 0.57 ± 0.03 | 2013 |
| | | GRIN | PI 170618 | Turkey | 0.94 ± 0.07 | 2013 |
| *Secale strictum* (C. Presl) C. | Other wild | GRIN | PI 383756 | Turkey | 5.51 ± 0.22 | 2011 |
| Presl | | GRIN | PI 240286 | Turkey | 18.12 ± 0.46 | 2011 |
| *Secale vavilovii* Grossh. | Putative | GRIN | PI 284842 | Hungary | 28.81 ± 0.72 | 2011 |
| | progenitor | GRIN | PI 573649 | Afghanistan | 21.74 ± 0.63 | 2011 |
| *Stipa capensis* Thunb. | Other wild | GRIN | PI 170808 | Turkey | 1.31 ± 0.38 | 2013 |
| | | GRIN | PI 226672 | Iran | 1.49 ± 0.21 | 2013 |
| *Taeniatherum caput-medusae* | Other wild | GRIN | PI 577709 | Turkey | 4.13 ± 0.11 | 2011 |
| (L.) Nevski | | GRIN | PI 577710 | Turkey | 7.80 ± 1.90 | 2011 |
| | | GRIN | PI 227665 | Iran | 6.80 ± 0.38 | 2013 |
| | | GRIN | PI 561095 | Turkey | 3.59 ± 0.12 | 2013 |
| *Triticum araraticum* Jakubz. | Putative | JIC | 1150001 | USSR | 18.03 ± 2.01 | 2013 |
| | progenitor | JIC | 1150002 | Azerbaijan | 35.15 ± 4.39 | 2013 |

| | | | | | | |
|---|---|---|---|---|---|---|
| *Triticum monococcum* L. subsp. *aegilopoides* (Link) Thell. | Confirmed progenitor | GRIN | PI 427452 | Turkey | 14.51 ± 1.39 | 2011 |
| | | GRIN | PI 245726 | Turkey | 16.13 ± 2.25 | 2011 |
| | | GRIN | PI 554577 | Turkey | 13.41 ± 2.43 | 2013 |
| | | GRIN | PI 427997 | Lebanon | 14.97 ± 1.25 | 2013 |
| | | IPK | TRI17105 | Iran | 20.26 ± 0.61 | 2013 |
| *Triticum turgidum* L. subsp. *dicoccoides* (Körn. ex Asch. & Graebn.)Thell. | Confirmed progenitor | GRIN | PI 300989 | Israel | 27.28 ± 1.04 | 2011 |
| | | GRIN | PI 428022 | Turkey | 23.23 ± 0.98 | 2011 |
| | | GRIN | PI 352324 | Lebanon | 26.71 ± 1.09 | 2013 |
| | | GRIN | PI 428017 | Turkey | 43.11 ± 4.33 | 2013 |
| | | GRIN | PI 487242 | Syria | 27.55 ± 2.50 | 2013 |
| *Triticum urartu* Tumanian ex Gandilyan | Putative progenitor | JIC | 1010004 | Turkey | 16.36 ± 1.10 | 2013 |
| | | JIC | 1010042 | Iran | 19.05 ± 2.22 | 2013 |
| | | JIC | 1010044 | Lebanon | 10.68 ± 1.22 | 2013 |

Seed banks used were the USDA Germplasm Resources Information System (GRIN), IPK Gatersleben Genebank (IPK), John Innes Centre Germplasm Resources Unit (JIC) and Kew Royal Botanic Gardens' Millennium Seed Bank (Kew).

**Table S2** List of legume accessions used, including the seed bank it was obtained from, country of origin and mean individual seed mass in mg (± 1SE)

| Species | Group | Seed bank | Accession | Country of origin | Mean seed mass (mg) (± 1SE) | Experiment |
|---|---|---|---|---|---|---|
| *Cicer judaicum* Boiss. | Other wild | GRIN | PI 510662 | Israel | 18.26 ± 0.03 | 2011 |
| | | GRIN | PI 593710 | Syria | 17.9 ± 0.39 | 2011 &2013 |
| | | GRIN | PI 510659 | Israel | 23.7 ± 0.89 | 2013 |
| | | GRIN | PI 599078 | Jordan | 24.08 ± 1.67 | 2013 |
| *Cicer reticulatum* Ladiz. | Confirmed Progenitor | GRIN | PI 489777 | Turkey | 127.2 ± 5.41 | 2011 & 2013 |
| | | GRIN | PI 510656 | Turkey | 141.0 ± 4.51 | 2011 |
| | | GRIN | PI 599092 | Turkey | 156.69 ± 15.32 | 2013 |
| *Coronilla scorpioides* (L.) W. D. J. Koch | Other wild | Kew | 25225 | Greece | 2.23 ± 0.12 | 2013 |
| *Lathyrus aphaca* L. | Other wild | GRIN | W6 2747 | Syria | 40.70 ± 4.85 | 2013 |
| | | GRIN | PI 227511 | Iran | 21.13 ± 1.75 | 2013 |
| | | GRIN | PI 358822 | Israel | 18.33 ± 4.44 | 2013 |
| *Lathyrus cicera* L. | Other wild | GRIN | PI 174236 | Turkey | 88.36 ± 16.86 | 2013 |
| | | GRIN | PI 237639 | Syria | 77.46 ± 10.35 | 2013 |
| | | GRIN | PI 250758 | Iran | 56.15 ± 5.46 | 2013 |
| *Lathyrus inconspicuus* | Other wild | Kew | 10975 | Greece | 23.79 ± 1.26 | 2013 |
| *Lens culinaris* Medik subsp. *odemensis* (Ladiz.) | Other wild | GRIN | PI 572364 | Turkey | 8.6 ± 0.26 | 2011 & 2013 |
| | | GRIN | PI 572360 | Israel | 16.5 ± 0.25 | 2011 & 2013 |
| *Lens culinaris* subsp. *orientalis* (Boiss.) | Confirmed progenitor | GRIN | PI 572371 | Israel | 8.2 ± 0.25 | 2011 & 2013 |
| | | GRIN | PI 572379 | Turkey | 10.6 ± 0.24 | 2011 |
| | | GRIN | PI 572374 | Iran | 19.03 ± 1.56 | 2013 |
| | | GRIN | PI 572366 | Turkey | 8.71 ± 0.69 | 2013 |
| *Lens nigricans* (M. Bieb.) Godr. | Other wild | GRIN | PI 572359 | Turkey | 7.47 ± 0.37 | 2013 |
| | | GRIN | PI 615676 | Turkey | 7.79 ± 0.82 | 2013 |
| | | GRIN | PI 615677 | Turkey | 7.43 ± 0.74 | 2013 |
| *Lupinus angustifolius* L. | Other wild | Kew | 78690 | Greece | 96.09 ± 5.67 | 2013 |

| | | | | | | |
|---|---|---|---|---|---|---|
| *Melilotus indicus* L. | Other wild | GRIN | MEL 22 | unknown | 0.56 ± 0.03 | 2013 |
| | | GRIN | MEL 28 | Egypt | 0.83 ± 0.06 | 2013 |
| *Medicago polymorpha* L. | Other wild | GRIN | W6 2092 | Turkey | 5.53 ± 0.53 | 2013 |
| | | GRIN | W6 5311 | Lebanon | 3.43 ± 0.44 | 2013 |
| | | GRIN | W6 5332 | Turkey | 3.18 ± 0.34 | 2013 |
| *Pisum fulvum* Sm. | Other wild | GRIN | PI 560065 | Israel | 67.2 ± 3.79 | 2011 |
| | | GRIN | PI 595946 | Turkey | 48.2 ± 1.99 | 2011 & 2013 |
| | | JIC | 2514 | Syria | 73.77 ± 4.25 | 2013 |
| | | GRIN | PI 595944 | Israel | 62.85 ± 5.17 | 2013 |
| *Pisum sativum* L. subsp. *elatius* (Steven ex M. Bieb.) Asch. & Graebn. | Confirmed Progenitor | GRIN | PI 344004 | Turkey | 62.0 ± 1.65 | 2011 |
| | | GRIN | PI 343972 | Turkey | 87.7 ± 2.31 | 2011 |
| *Pisum sativum* L. subsp.*elatius* (Steven ex M. Bieb.) Asch. & Graebn. var. *pumilio* Meikle | Confirmed progenitor | GRIN | W6 2107 | Turkey | 120.7 ± 5.68 | 2011 |
| | | GRIN | W6 12613 | Turkey | 76.4 ± 3.08 | 2011 |
| | | GRIN | W6 2101 | Turkey | 70.29 ± 6.14 | 2013 |
| | | JIC | 3273 | Israel | 82.6 ± 4.79 | 2013 |
| | | JIC | 3274 | Israel | 72.9 ± 2.63 | 2013 |
| *Scorpiurus muricatus* L. | Other wild | GRIN | W6 2758 | Syria | 15.6 ± 1.26 | 2013 |
| | | GRIN | PI 226484 | Iran | 9.33 ± 1.07 | 2013 |
| | | GRIN | PI 330680 | Israel | 15.23 ± 1.19 | 2013 |
| *Vicia ervilia* (L.) Willd. | Confirmed Progenitor | GRIN | PI 229733 | Iran | 36.2 ± 1.42 | 2011 |
| | | GRIN | PI 206489 | Turkey | 41.8 ± 1.34 | 2011 |
| *Vicia narbonensis* L. | Other wild | GRIN | PI 294300 | Israel | 62.5 ± 15.36 | 2011 |
| | | GRIN | PI 206927 | Turkey | 206.8 ± 8.50 | 2011 |
| | | GRIN | PI 294298 | Israel | 91.5 ± 8.60 | 2013 |
| | | GRIN | PI 466295 | Israel | 151.25 ± 38.70 | 2013 |
| *Vicia peregrina* L. | Other wild | GRIN | PI 227472 | Iran | 48.83 ± 4.53 | 2013 |
| | | GRIN | PI 343956 | Turkey | 38.11 ± 4.34 | 2013 |
| | | GRIN | PI 407641 | Turkey | 54.47 ± 4.67 | 2013 |

**Table S3** Sample ubiquity (number of samples in which taxon occurs) list for the grass taxa used in this study, acquired from the archaeobotanical database

| Site | Total samples | *Hordeum spontaneum* | *Phalaris* | *Phalaris paradoxa* | *Triticum boeoticum/Secale montanum/vavilovii* | *Stipa* | *Taeniatherum caput-medusae* | *Triticum boeoticum* | *Triticum dicoccoides* |
|---|---|---|---|---|---|---|---|---|---|
| Abu Hureyra | 42 | 0 | 0 | 0 | 21 | 21 | 0 | 0 | 0 |
| Demirkoy | 8 | 5 | 0 | 0 | 1 | 0 | 2 | 0 | 0 |
| Douara Cave | 0 | 0 | 0 | 0 | 0 | 0 | 0 | 0 | 0 |
| el-Hemmeh | 3 | 1 | 1 | 0 | 0 | 1 | 0 | 0 | 0 |
| Gesher | 1 | 1 | 0 | 0 | 0 | 0 | 0 | 0 | 0 |
| Gesher Benot Yaaqov | 0 | 0 | 0 | 0 | 0 | 0 | 0 | 0 | 0 |
| Gilgal | 0 | 0 | 0 | 0 | 0 | 0 | 0 | 0 | 0 |
| Göbekli Tepe | 2 | 1 | 0 | 0 | 1 | 0 | 0 | 0 | 0 |
| Hallan Çemi | 179 | 49 | 0 | 0 | 13 | 41 | 76 | 0 | 0 |
| Hayonim Cave | 0 | 0 | 0 | 0 | 0 | 0 | 0 | 0 | 0 |
| Iraq ed-Dubb | 21 | 9 | 10 | 0 | 1 | 0 | 0 | 1 | 0 |
| Jerf el Ahmar | 538 | 258 | 0 | 0 | 233 | 14 | 1 | 32 | 0 |
| Jericho | 0 | 0 | 0 | 0 | 0 | 0 | 0 | 0 | 0 |
| Kebara Cave | 1 | 1 | 0 | 0 | 0 | 0 | 0 | 0 | 0 |
| Kortik Tepe | 2 | 0 | 0 | 0 | 1 | 0 | 1 | 0 | 0 |
| Mlefaat | 8 | 0 | 0 | 0 | 4 | 0 | 4 | 0 | 0 |
| Mureybet | 32 | 31 | 0 | 0 | 1 | 0 | 0 | 0 | 0 |
| Nahal Oren | 0 | 0 | 0 | 0 | 0 | 0 | 0 | 0 | 0 |
| Nemrik 9 | 0 | 0 | 0 | 0 | 0 | 0 | 0 | 0 | 0 |
| Netiv Hagdud | 17 | 8 | 1 | 0 | 0 | 8 | 0 | 0 | 0 |
| Ohalo II | 4 | 4 | 0 | 0 | 0 | 0 | 0 | 0 | 0 |
| Qermez Dere | 37 | 18 | 0 | 0 | 15 | 1 | 3 | 0 | 0 |
| Tell Abr | 60 | 17 | 0 | 0 | 25 | 5 | 0 | 13 | 0 |
| Tell Qaramel | 179 | 40 | 1 | 0 | 50 | 13 | 0 | 75 | 0 |
| Wadi al-Hammeh 27 | 5 | 3 | 0 | 0 | 0 | 2 | 0 | 0 | 0 |

| | | | | | | | | | |
|---|---|---|---|---|---|---|---|---|---|
| Wadi el-Jilat 6 | 1 | 0 | 0 | 0 | 0 | 1 | 0 | 0 | 0 |
| Wadi Faynan 16 | 1 | 0 | 0 | 0 | 0 | 1 | 0 | 0 | 0 |
| Zahrat adh-Dhra 2 | 23 | 15 | 0 | 0 | 0 | 8 | 0 | 0 | 0 |

**Table S4** Sample ubiquity (number of samples in which taxon occurs) list for the legume taxa used in this study, acquired from the archaeobotanical database

| Site | Total samples | *Cicer* | *Lens* | *Pisum elatius/sativum* | *Vicia* spp. excluding *V. ervilia* | *Vicia ervilia* |
|---|---|---|---|---|---|---|
| Abu Hureyra | 10 | 0 | 8 | 0 | 0 | 2 |
| Demirkoy | 1 | 0 | 0 | 1 | 0 | 0 |
| Douara Cave | 0 | 0 | 0 | 0 | 0 | 0 |
| el-Hemmeh | 3 | 1 | 1 | 0 | 1 | 0 |
| Gesher | 2 | 0 | 0 | 0 | 2 | 0 |
| Gesher Benot Yaaqov | 0 | 0 | 0 | 0 | 0 | 0 |
| Gilgal | 0 | 0 | 0 | 0 | 0 | 0 |
| Göbekli Tepe | 1 | 0 | 1 | 0 | 0 | 0 |
| Hallan Çemi | 75 | 0 | 11 | 41 | 0 | 23 |
| Hayonim Cave | 1 | 0 | 0 | 1 | 0 | 0 |
| Iraq ed-Dubb | 7 | 0 | 5 | 0 | 2 | 0 |
| Jerf el Ahmar | 292 | 0 | 228 | 5 | 58 | 54 |
| Jericho | 2 | 1 | 1 | 0 | 0 | 0 |
| Kebara Cave | 12 | 1 | 4 | 0 | 4 | 3 |
| Kortik Tepe | 0 | 0 | 0 | 0 | 0 | 0 |
| Mlefaat | 8 | 0 | 4 | 0 | 0 | 4 |
| Mureybet | 34 | 0 | 16 | 13 | 2 | 3 |
| Nahal Oren | 3 | 0 | 0 | 0 | 3 | 0 |
| Nemrik 9 | 0 | 0 | 0 | 0 | 0 | 0 |
| Netiv Hagdud | 8 | 0 | 7 | 0 | 0 | 1 |
| Ohalo II | 1 | 0 | 1 | 0 | 0 | 0 |
| Qermez Dere | 31 | 0 | 27 | 0 | 1 | 3 |
| Tell Abr | 22 | 0 | 22 | 0 | 0 | 0 |
| Tell Qaramel | 102 | 0 | 85 | 2 | 9 | 6 |

| | | | | | | |
|---|---|---|---|---|---|---|
| Wadi al-Hammeh 27 | 1 | 0 | 1 | 0 | 0 | 0 |
| Wadi el-Jilat 6 | 0 | 0 | 0 | 0 | 0 | 0 |
| Wadi Faynan 16 | 0 | 0 | 0 | 0 | 0 | 0 |
| Zahrat adh-Dhra 2 | 13 | 0 | 13 | 0 | 0 | 0 |

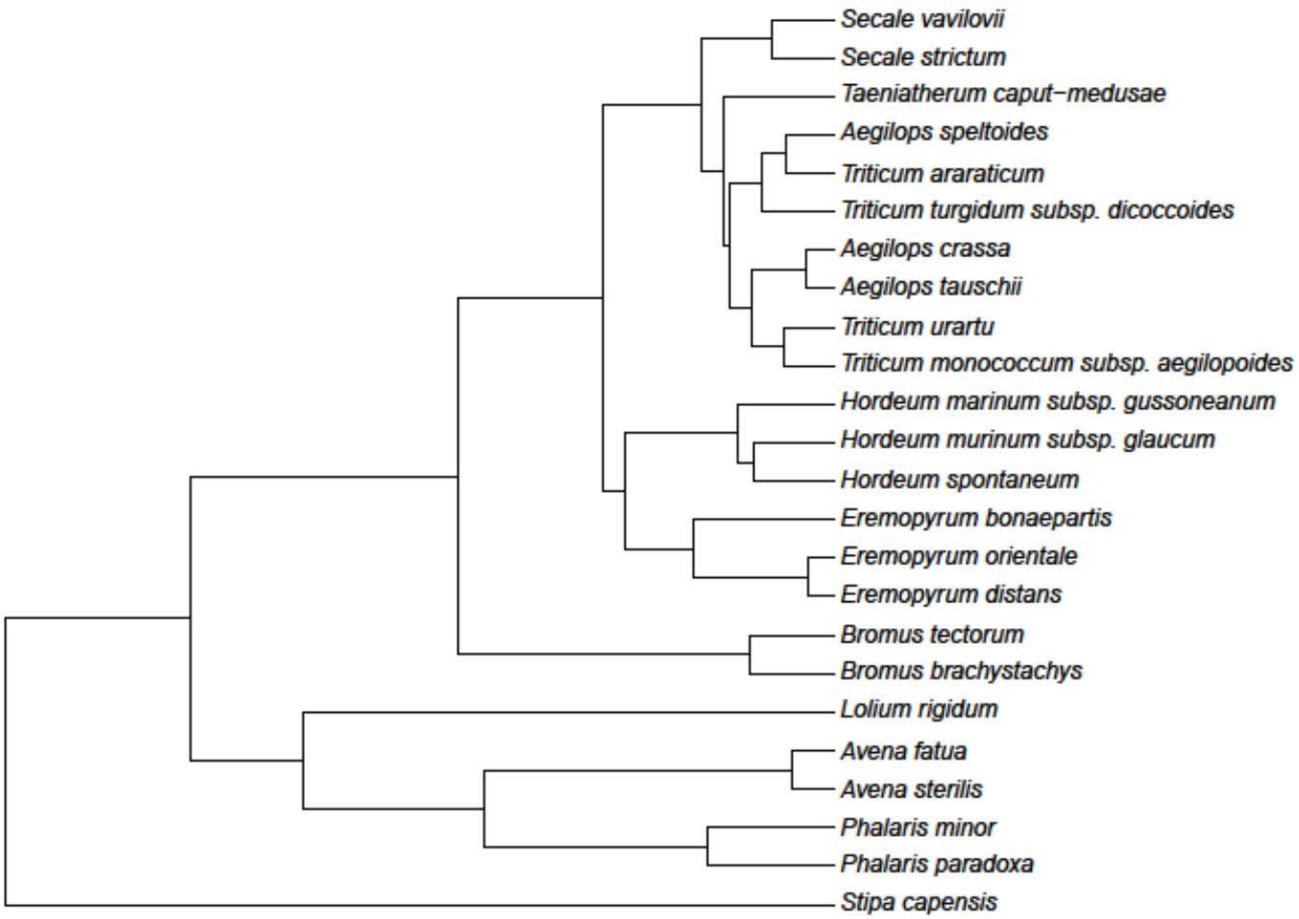


**Fig. S1** Grass phylogeny for the 24 species used in our experiments, based on the two plastid markers *ndhF* and *trnKmatK*.

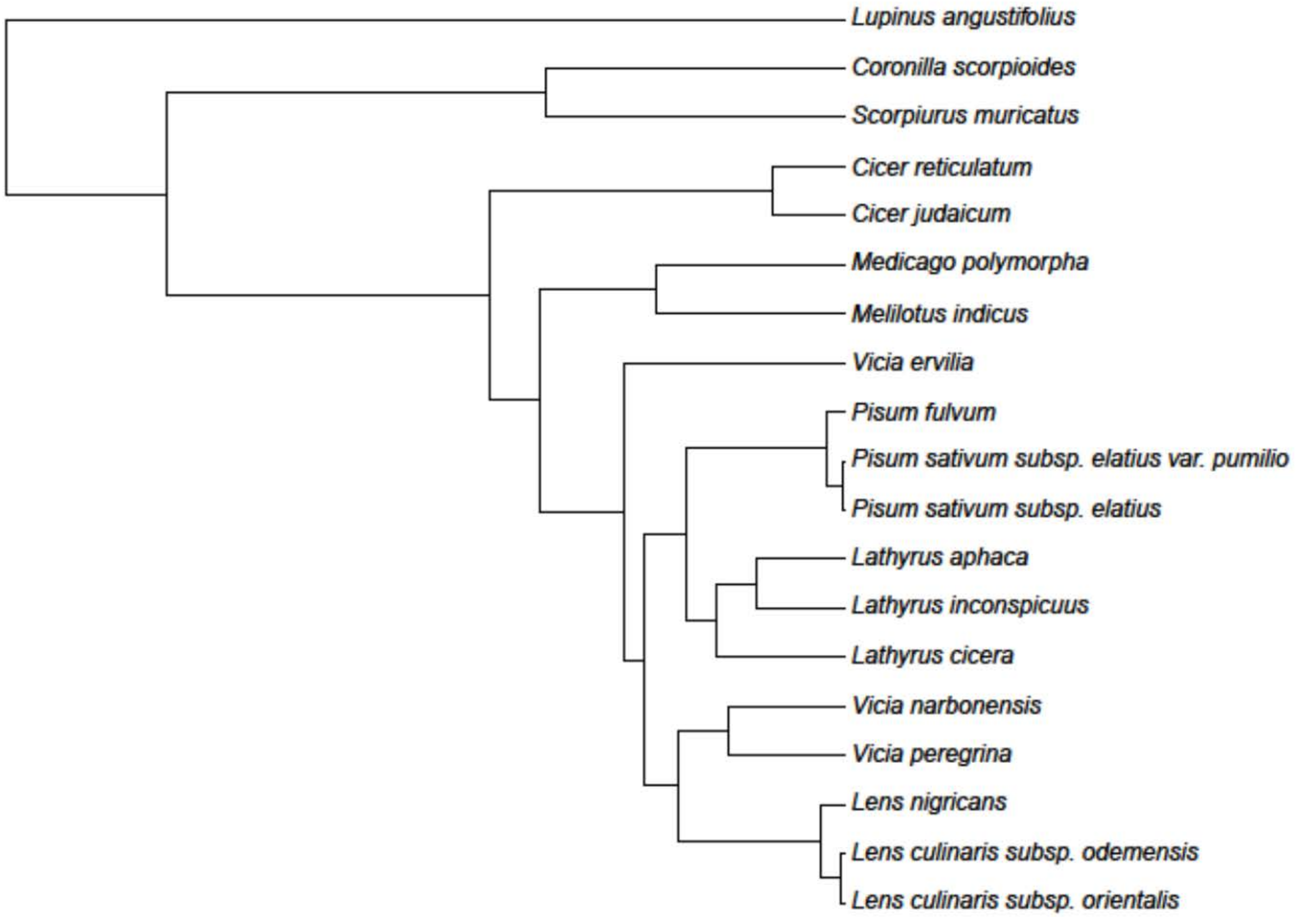


**Fig. S2** Legume phylogeny for the 19 species used in our experiments, based on the plastid marker *trnKmatK*.

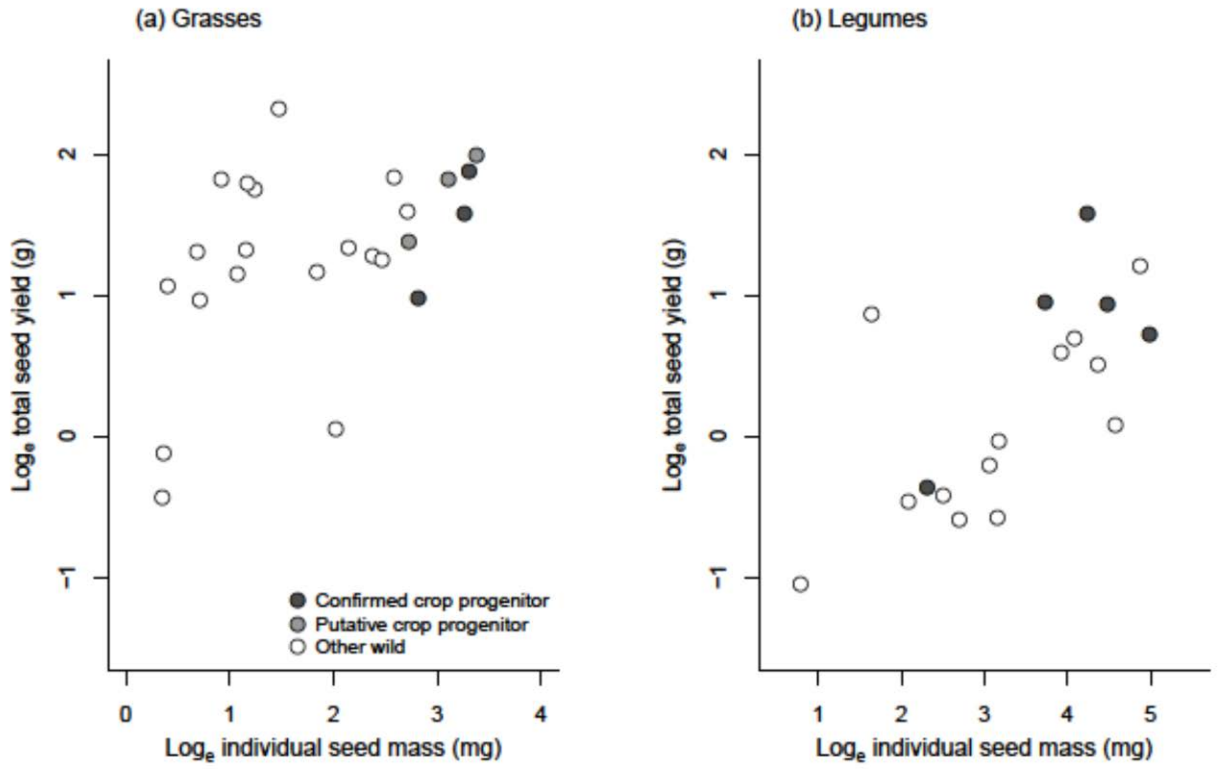


**Fig. S3** Relationship between total seed yield and individual seed mass for (a) grasses and (b) legumes. Points are species means and are colour coded by category: dark grey circles, confirmed crop progenitors; light grey circles, putative crop progenitors; white circles, other wild species. Both axes are shown on a log$_e$ scale. There was no correlation between these two traits for grasses, but there was a positive correlation for legumes ($P < 0.001$).

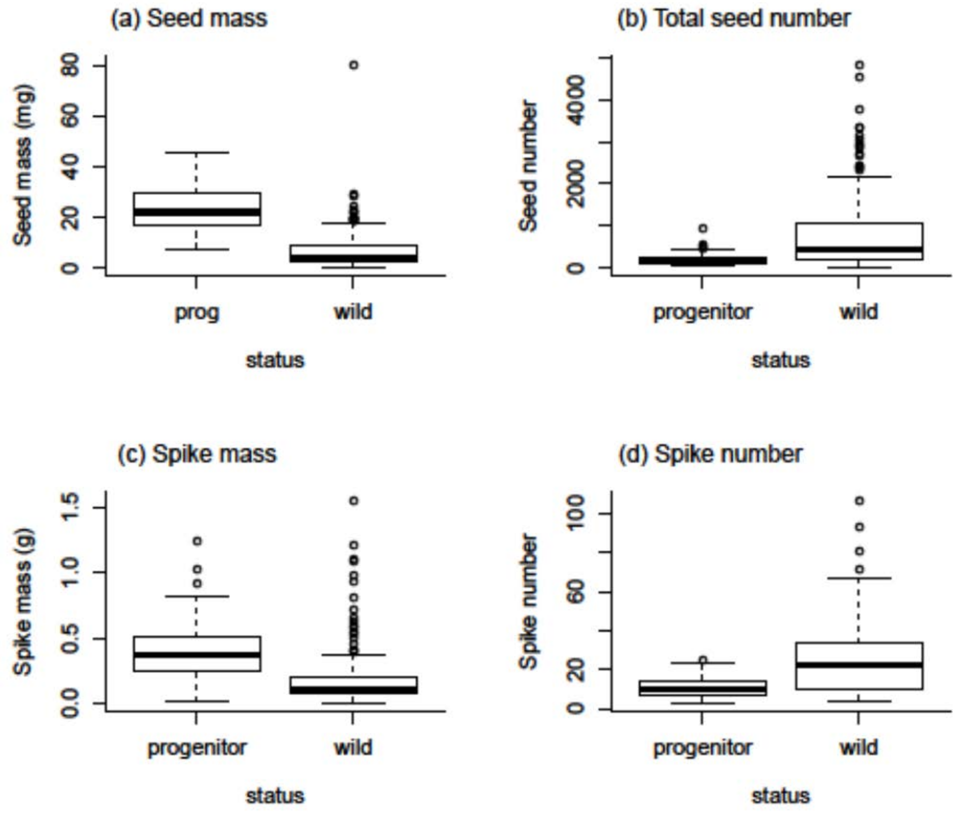


**Fig. S4** Variability of traits relating to total seed yield in grasses, using the longlist of crop progenitors. Boxplots of (a) individual seed mass (mg), (b) total seed number per plant, (c) spike mass (g) and (d) spike number per plant are shown. Error bars represent 1.5 × the interquartile range.

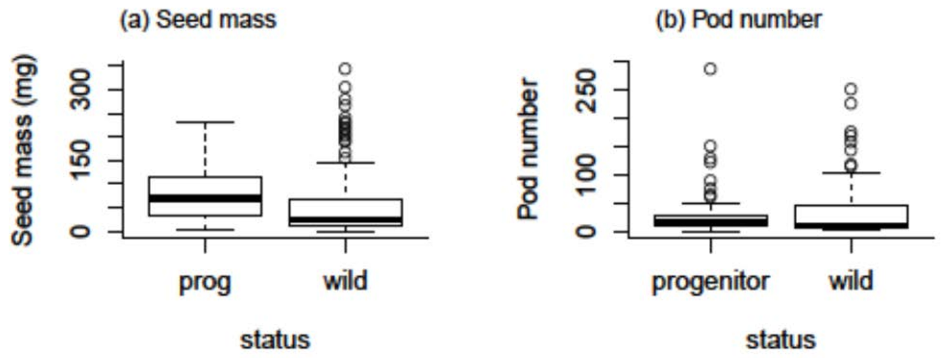


**Fig. S5** Variability of traits relating to total seed yield in legumes. Boxplots of (a) individual seed mass (mg) and (b) pod number per plant are shown. Error bars represent 1.5 × the interquartile range.
